# Mineralocorticoid Receptor in Myeloid Cells Mediates Angiotensin II-Induced Vascular Dysfunction in Female Mice

**DOI:** 10.3389/fphys.2021.588358

**Published:** 2021-03-29

**Authors:** Camila Manrique-Acevedo, Jaume Padilla, Huma Naz, Makenzie L. Woodford, Thaysa Ghiarone, Annayya R. Aroor, Jack L. Hulse, Francisco J. Cabral-Amador, Vanesa Martinez-Diaz, Chetan P. Hans, Adam Whaley-Connell, Luis A. Martinez-Lemus, Guido Lastra

**Affiliations:** ^1^Division of Endocrinology and Metabolism, Department of Medicine, University of Missouri, Columbia, MO, United States; ^2^Dalton Cardiovascular Research Center, University of Missouri, Columbia, MO, United States; ^3^Research Service, Harry S. Truman Memorial Veterans’ Hospital, Columbia, MO, United States; ^4^Department of Nutrition and Exercise Physiology, University of Missouri, Columbia, MO, United States; ^5^Division of Cardiovascular Medicine, Department of Medicine, University of Missouri, Columbia, MO, United States; ^6^Division of Nephrology and Hypertension, Department of Medicine, University of Missouri, Columbia, MO, United States; ^7^Department of Biomedical, Biological and Chemical Engineering, University of Missouri, Columbia, MO, United States; ^8^Department of Medical Pharmacology and Physiology, University of Missouri, Columbia, MO, United States

**Keywords:** arterial stiffening, macrophage, endothelium, inflammation, adipose tissue

## Abstract

Enhanced mineralocorticoid receptor (MR) signaling is critical to the development of endothelial dysfunction and arterial stiffening. However, there is a lack of knowledge about the role of MR-induced adipose tissue inflammation in the genesis of vascular dysfunction in women. In this study, we hypothesize that MR activation in myeloid cells contributes to angiotensin II (Ang II)-induced aortic stiffening and endothelial dysfunction in females *via* increased pro-inflammatory (M1) macrophage polarization. Female mice lacking MR in myeloid cells (MyMRKO) were infused with Ang II (500 ng/kg/min) for 4 weeks. This was followed by determinations of aortic stiffness and vasomotor responses, as well as measurements of markers of inflammation and macrophage infiltration/polarization in different adipose tissue compartments. MyMRKO mice were protected against Ang II-induced aortic endothelial stiffening, as assessed *via* atomic force microscopy in aortic explants, and vasorelaxation dysfunction, as measured by aortic wire myography. In alignment, MyMRKO mice were protected against Ang II-induced macrophage infiltration and M1 polarization in visceral adipose tissue (VAT) and thoracic perivascular adipose tissue (tPVAT). Collectively, this study demonstrates a critical role of MR activation in myeloid cells in the pathogenesis of vascular dysfunction in females associated with pro-inflammatory macrophage polarization in VAT and tPVAT. Our data have potential clinical implications for the prevention and management of cardiovascular disease in women, who are disproportionally at higher risk for poor outcomes.

## Introduction

Chronic inflammation is invariably present in the setting of cardiovascular disease (CVD) (Saltiel and Olefsky, 2017). In different models of renin–angiotensin–aldosterone system (RAAS) overactivation, inflammation promotes hypertrophy, remodeling, and dysfunction of cardiovascular tissue that ultimately results in clinical CVD (Saltiel and Olefsky, 2017). These conditions are also associated with inflammation of the adipose tissue (Brown et al., 2014). In turn, inflammation in fat tissue encompasses a distinctive macrophage infiltration and polarization pattern, characterized by a pro-inflammatory M1 phenotype at the expense of anti-inflammatory M2 macrophages (Kraakman et al., 2014; Lee et al., 2015). Activated immune cells promote oxidative stress in vascular tissues and contribute to the genesis of endothelial dysfunction and vascular stiffening (van Bussel et al., 2011; Aroor et al., 2013; Jain et al., 2014). These changes precede the development of hypertension and CVD (Kaess et al., 2012; Weisbrod et al., 2013).

Visceral adipose tissue (VAT) depots have been extensively associated with heightened CVD risk (Goodpaster et al., 2003; Klein, 2004; Fox et al., 2007). However, there is also growing interest in perivascular adipose tissue (PVAT) as it exhibits unique features that differentiate it from VAT or subcutaneous adipose tissue and has been shown to modulate the function of underlying blood vessels (Lastra and Manrique, 2015). Clinical studies have reported that dysfunctional PVAT contributes to increased risk for CVD independently of VAT (Lastra and Manrique, 2015). Enhanced RAAS activation impairs the normal vasodilatory and anti-contractile properties of PVAT, leading to inflammation, vasoconstriction, and vascular remodeling (Agabiti-Rosei et al., 2018; Dos Reis Costa et al., 2020).

RAAS activation leads to augmented mineralocorticoid receptor (MR) signaling (Lastra et al., 2008). MR is expressed in immune cells (Muñoz-Durango et al., 2015), and its stimulation leads to inflammation, oxidative stress, and vascular remodeling (Jaisser and Farman, 2016; Tesch and Young, 2017). Accordingly, MR deletion in myeloid cells has been shown to prevent macrophage polarization, cardiovascular fibrosis, and remodeling in male mice hypertensive models (Rickard et al., 2009; Usher et al., 2010; Shukri et al., 2018).

In the vasculature, mineralocorticoids elicit dimorphic responses that impact females to a greater extent compared to males (Mehta et al., 2016). Indeed, obese and insulin-resistant women display more frequent and aggressive frequency of CVD compared to men (Nayyar et al., 2018). Although available clinical and preclinical studies have established that MR activation is critical to the development of vascular dysfunction impacting females more severely than males, there is still a lack of knowledge about the relationship between MR activation in myeloid cells and the development of vascular/endothelial dysfunction in females. In this study, we tested the hypothesis that MR activation in myeloid cells mediates aortic stiffening and endothelial dysfunction induced by a continuous infusion of angiotensin II (Ang II) in female mice, and that these vascular effects parallel with changes in adipose tissue macrophage infiltration and pro-inflammatory M1 polarization.

## Materials and Methods

### Animals

Animal procedures were performed in accordance with the Institutional Animal Care and Use Committee (IACUC) at the University of Missouri-Columbia and National Institutes of Health guidelines. The IACUC at the University of Missouri-Columbia reviewed and approved the animal protocol followed. Mice used in these experiments were harbored under a 12-h/day illumination regimen and fed with standard mouse chow and water for *ad libitum* consumption. Double floxed MR (MRFL2) mice were kindly provided by Dr. Richard Mortensen from the University of Michigan, and this model has been previously characterized (Berger et al., 2006; Usher et al., 2010). Briefly, MRFl2 were crossed with LysM-Cre mice (endogenous M lysozyme driving expression of Cre-recombinase, The Jackson Laboratory) in order to obtain myeloid-specific MR knockout mice (MRFl2/LysM-Cre+). Herein, we refer to MRFl2/LysM-Cre+ as “MyMRKO” and MR intact (MRFl2/LysM-Cre−) as littermate (LM) controls. We used ear punch and genotyping to detect the presence of both the floxed and deleted alleles, as previously described (Berger et al., 2006; Usher et al., 2010). Mice were infused with Ang II (500 ng/kg/min) or saline for 4 weeks, as previously described in our laboratory (Brown et al., 2017). Both treatments were administered by micro-osmotic pump Model 1004 (ALZET, Cupertino, CA) located subcutaneously between the scapulae. Three cohorts of female mice were studied: LM-saline, LM-Ang II, and MyMRKO-Ang II. Separate cohorts of LM-Ang II and MyMRKO-Ang II mice were devoted to assessing macrophage infiltration and polarization in fat. A saline-infused MyMRKO cohort was not included in this investigation, as it has been previously reported that these mice do not exhibit phenotypical variations in cardiovascular tissue or macrophage infiltration/polarization under control conditions (Rickard et al., 2009; Usher et al., 2010).

Plasma insulin concentrations were determined using a commercially available, mouse-specific ELISA (Alpco Diagnostics, Salem, NH). Aldosterone levels were analyzed by radioimmunoassay at Michigan State University Diagnostic Veterinary Laboratory.

### Blood Pressure

Systolic blood pressure in mice was determined non-invasively using a CODA tail-cuff blood pressure system (CODA-HT2; Kent Scientific, Torrington, CT) within 1 week of killing as previously described (Grunewald et al., 2019). Animals were acclimated to the restraints and tail-cuffs for a minimum of three consecutive days prior to blood pressure determination. A minimum of eight blood pressure readings were averaged for each animal.

### Aortic Stiffness

Atomic force microscopy (AFM) was used to assess *ex vivo* endothelial stiffness in enface aortic preparations as previously described (Padilla et al., 2019). Perivascular fat and connective tissue were gently removed from the aortic explants as the piece of aorta needs to lay flat on a hard surface to be probed with the AFM cantilever. A total of at least 10 curves were obtained per cell/aortic explant site and repeated for at least four cells/sites per sample. Elastic moduli were calculated from the force curves by fitting them to the Hertz model of a conical tip using a custom-made Python script as previously described (Hussain et al., 2014).

### *Ex vivo* Aortic Vasomotor Function

Thoracic aortic 2-mm rings, cleaned of perivascular fat and connective tissue, were mounted in wire myograph organ bath chambers (620M, Danish Myo Technology, Hinnerup, Denmark) containing warmed physiological saline solution gassed with 95% O_2_–5% CO_2_ and maintained at 37°C, as previously described (Grunewald et al., 2019; Padilla et al., 2019). Arterial rings were constricted with KCl (80 mM) to assess viability. After washout, aortas were then preconstricted with the prostaglandin H2/thromboxane A2 receptor agonist, U-46619 (20 nM). Relaxation of arterial rings to acetylcholine (ACh, 10^–9^ to 10^–5^ M), the NO-donor sodium nitroprusside (SNP, 10^–9^ to 10^–4^ M), and insulin (Novolin R, Novo Nordisk; 10^–9^ to 10^–5^ M) was assessed by cumulative addition of agonist to the vessel bath. Endothelium-dependent relaxation was assessed using ACh and insulin, while SNP assessed endothelium-independent relaxation. Area under the curve (AUC) of each concentration–response curve was calculated using the trapezoidal rule.

### RNA Isolation

To perform relative quantification by Real-Time PCR (RT-PCR), total RNA was isolated from thoracic (tPVAT) and abdominal (aPVAT) aortic PVAT as well as 30–41 mg of perigonadal fat, which is widely considered to be VAT in rodents (Bjørndal et al., 2011; Chusyd et al., 2016), per animal. After dissection, 1 ml of QIAzol Lysis Reagent (Qiagen, Hilden, Germany) was added to each sample. Samples were stored at −80°C until RNA isolation. Tissues were disrupted and homogenized in intervals of 2 min at 20 1/s by Tissulyser (Qiagen, Hilden, Germany) followed by 5 min of cooling on ice. This process was repeated three times. Then, RNA was concentrated and precipitated using RNeasy Lipid Tissue MiniKit (Qiagen, Hilden, Germany), following its commercial standard protocol. The optional digestion step with DNase, recommended by the protocol, was also performed using RNase-Free DNase Set (Qiagen, Hilden, Germany). DNase treatment was performed with 10 μl of DNase I (27.27 Kunitz units) for 15 min at room temperature. Total RNA from tPVAT, aPVAT, and perigonadal adipose tissue (PGAT) was resuspended in 15 and 30 μl of ultrapure water. The concentration and quality of RNA were measured using NanoDrop ND-1000 spectrophotometer (Thermo Fisher Scientific, Wilmington, DE). First-strand cDNA was synthesized from total RNA using the ImProm-II Reverse Transcription System kit (Promega, Madison, WI).

### Real-Time PCR

Markers of inflammation and macrophage polarization were determined by relative quantification with RT-PCR on a CFX96 Touch Real Time PCR Detection System (Bio-Rad, Hercules, CA) using the iTaq Universal SYBR Green Supermix (Bio-Rad Laboratories, Hercules, CA) according to the manufacturer’s instructions. Each experiment was performed with two technical replicates containing 12.5, 15, and 20 ng of complementary DNA of tPVAT, aPVAT, and PGAT, respectively, 10 μl iTaq Universal SYBR Green Supermix (2X), and forward and reverse primers (10 pM/ml; Integrated DNA Technologies, San Diego, CA). The primer sequences used are listed in Table 1. A negative control was included for each primer pair in accordance with MIQE guidelines (Bustin et al., 2009). RT-PCR reaction conditions were as follows: one cycle of 95°C for 2 min, followed by 39 cycles of 95°C for 5 s, 60°C for 30 s, and one cycle more of 95°C for 5 s. In order to check the specificity of the primers, a melting curve analysis for each amplicon was performed at the end of each run with a temperature gradient from 65 to 95°C with an increment of 0.5°C s^–1^. Bio-Rad CFX manager software was used to obtain Cq value. Glyceraldehyde 3-phosphate dehydrogenase (GAPDH) was used as a housekeeping control gene. Calculations of gene expression were done using the 2^–Δ^ ^Δ^ ^Ct^ method and presented as fold difference compared with the LM-Saline group, which was set to 1. In order to standardize experimental protocols and their information for publication, these experiments adopted the Minimum Information for Publication of Quantitative Real-time PCR Experiments (MIQE) guidelines, which promote consistency between laboratories and increase experimental transparency (Bustin et al., 2009).

**TABLE 1:** Sequences of primers used in this investigation.

Gene studied	Primer sequence
CD11b	Forward: 5′-CCA AGA CGA TCT CAG CAT-3′ Reverse: 5′-TTC TGG CTT GCT GAA TCC TT-3′
CD206	Forward: 5′-CAA GGA AGG TTG GCA TTT GT-3′ Reverse: 5′- CCT TTC AGT CCT TTG CAA GC-3′
GAPDH	Forward: 5′-GGA GAA ACC TGC CAA GTA TGA-3′ Reverse: 5′- TCC TCA GTG TAG CCC AAG A-3′
CD11c	Forward: 5′-ACA CAG TGT GCT CCA GTA TGA-3 ′ Reverse: 5′-GCC CAG GGA TAT GTT CAC AGC-3′
CD68	Forward: 5′-CCC AAT TCA GGG TGG AAG AA-3′ Reverse: 5′ATC CAA AGG TAA GCT GTC GAT AA-3′
TNF-a	Forward: 5′-GCC TCT TCT CAT TCC TGC TTG-3′ Reverse: 5′-CTG ATG AGA GGG AGG CCA TT-3′
IL-6b	Forward: 5′-GAT AAG CTG GAG TCA CAG AAG G-3′ Reverse: 5′-TTG CCG AGT AGA AGA TCT CAA AGT G-3′
IL-10	Forward: 5′-CCA AGC CTT ATC GGA AAT GA-3′ Reverse: 5′-TTT TCA CAG GGG AGA AAT CG-3′
MCP-1	Forward: 5′-GTC TCA ACC AGA TGC AGT TAA T-3′ Reverse: 5′-CTG CTG GTG ATT CTC TTG TAG TT-3′
N0 × 2	Forward: 5′-CTT TGG TAC AGC CAG TGA AGA-3′ Reverse: 5′-CCA GAC AGA CTT GAG AAT GGA G-3′
N0 × 4	Forward: 5′ CTG GAC CTT TGT GCC TTT ATT G-3′ Reverse: 5’-AGG GAT GAT TGA TGA CTG AGA TG-3’

### Single-Cell Preparation From Adipose Tissue

Visceral fat pads from the different cohorts were dissected and placed in buffer containing Hanks’ balanced salt solution (HBSS) + 10% fetal bovine serum (FBS). Fat pads were cut and minced in digestion buffer [HBSS, 20 mm 4-(2-hydroxyethyl)-1-piperazineethanesulfonic acid (HEPES), 1% bovine serum albumin (BSA), and 1 mg/ml collagenase type II] and incubated at 37°C for 45 min. Digested tissue was passed through a 70-μm cell strainer and centrifuged at 500 × g for 5 min. Pelleted cells were resuspended, and red blood cell lysis buffer was added and kept at room temperature for 2 min. Staining buffer was added and centrifuged at 500 × g for 5 min, and the cell pellet was resuspended in staining buffer and used for flow staining.

### Flow Cytometry

For antibody staining, 1 × 10^6^ cells/sample were used. Surface antibodies (1:50 dilution) PE anti-mouse CD45 (clone: 30-F11), APC/Cyanine7 anti-mouse F4/80 (clone: BM8), FITC anti-mouse CD11b (clone: M1/70), Pacific Blue^TM^ anti-mouse CD11c (clone: N418), PerCP/Cyanine5.5 anti-mouse CD206 (clone:C068C2), and PE/Cyanine7 anti-mouse I-A/I-E (MHC-II) (clone: M5/114.15.2) were added and incubated for 30 min at 4°C in the dark. After staining, cells were washed twice with staining buffer, and pellets were dissolved in 400 ml staining buffer before acquiring the samples. All the antibodies were obtained from BioLegend, San Diego, CA, United States. The data were collected using fluorescence activated cell sorting (FACS) Canto flow cytometer (BD Biosciences, Franklin Lakes, NJ, United States) and analyzed cytometry along with isotype controls using FlowJo software (Tarique et al., 2020).

### Histological Assessment

#### Macrophage Infiltration in Visceral Adipose Tissue

Formalin-fixed VAT samples were processed through paraffin embedding, sectioned at 5 μm, and stained with macrophage marker Mac-2 antibody (CL8942AP, 1:1,000; Cedarlane, Burlington, ON, CA), as previously described (Winn et al., 2019). Images were acquired with an Olympus IX51 microscope. Images were obtained by an investigator in a blinded manner, and objective quantification of macrophage infiltration was done by determining the positive Mac-2-stained area per ×10 fields of view using ImageJ software (NIH public domain; National Institutes of Health, Bethesda, MD). Crown-like structure density was defined as Mac-2-positive stained area (Huang et al., 2013). The average of three X10 fields of view was determined for each animal.

#### Aortic Inducible Nitric Oxide Synthase

Formalin-fixed abdominal aorta samples were processed through paraffin embedding, sectioned at 5 μm, and stained with inducible nitric oxide synthase (iNOS) antibody (ab 15323, 1:200; Abcam, Cambridge, MA, United States). Specificity of the antibody was confirmed using appropriate IgG controls (I-1000-5; Vector Laboratories, Burlingame, CA) in place of the primary antibodies at the same concentrations as previously described (Hans et al., 2012; Sharma et al., 2019). iNOS-positive myeloid and endothelial cells were counted (three equally sized regions of interest per slide) visually on the basis of nuclear shape and size at 40× and 100× magnification. The immunostaining patterns were examined by two investigators in a blinded manner.

### Statistical Analysis

Statistical analyses were performed using GraphPad Prism (version 8.2.0). All data were checked for normality by the Shapiro–Wilk test. Whenever data were not normally distributed, a logarithmic transformation was applied to normalize it. If transformations did not normalize data, we performed the equivalent non-parametric statistic test. Statistical comparisons were performed using one-way or two-way ANOVA followed by Tukey *post hoc* tests when applicable or Student’s *t*-test, as appropriate. Values are expressed as means ± standard error of the mean (SEM). Statistical significance was accepted at *p* ≤ 0.05.

## Results

**FIGURE 1:**
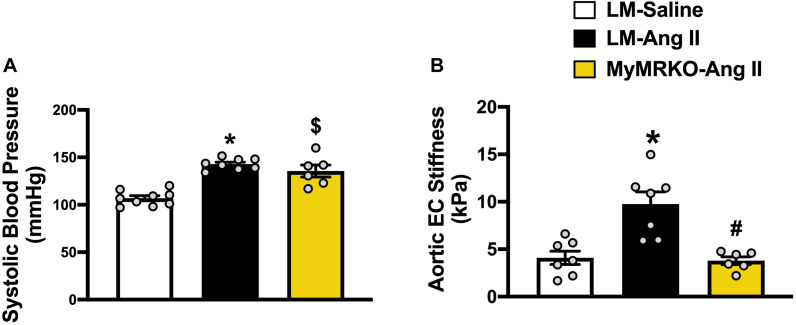
Ang II-induced aortic stiffening is ameliorated by myeloid cell MR deletion. Systolic blood pressure **(A)** and *ex vivo* assessment of aortic endothelial stiffness by atomic force microscopy **(B)**. *p ≤ 0.05 LM-Ang II vs. LM-Saline; ^#^*p* ≤ 0.05 LM-Ang II vs. MyMRKO-Ang II; ^$^*p* ≤ 0.05 MyMRKO-Ang II vs. LM-Saline. *n* = 6–9 for all groups. EC, endothelial cell; LM, littermate; Ang II, angiotensin II; MyMRKO, myeloid mineralocorticoid receptor knockout.

The average age of mice at the start of the experimental protocol was 22 ± 1 weeks. No differences in weight were found between the three cohorts (Table 2). We measured plasma aldosterone concentrations at the time of euthanasia in order to confirm the effect of Ang II infusion. As expected, aldosterone concentrations were significantly higher in Ang II-infused mice (Table 2). We also assessed insulin levels after a 5-h fast at the time of euthanasia. Our data did not reveal any significant differences across groups of mice (Table 2), thus suggesting that changes in neither body weight nor insulin concentrations account for the differences seen in vascular outcomes. Further, we assessed systolic blood pressure *via* the tail-cuff method. The 4-week infusion of Ang II resulted in a significant elevation in systolic blood pressure on both cohorts, and lack of MR in myeloid cells had no effect (Figure 1A).

**TABLE 2:** Body weight and insulin and plasma aldosterone levels in female MyMRKO and LM mice after treatment with Ang II (500 ng/kg/min) or saline *via* osmotic minipumps for 4 weeks.

	LM-Saline	LM-Ang II	MyMRKO-Ang II
Body weight (grams)	21.4 ± 0.6	20.8 ± 1.4	20.4 ± 1.9
Insulin (ng/dL)	0.51 ± 0.05	0.32 ± 0.22	0.58 ± 0.10
Aldosterone (pmol/L)	2317.94 ± 316.57	6407.60 ± 594.15*	6332.08 ± 447.07$

Samples were collected after 5-h fasting and prior to sacrifice. Results are presented as means ± standard error (SE), n = 7–15 for all groups. LM, littermates; Ang II, Angiotensin II; MyMRKO, myeloid mineralocorticoid receptor knockout.

*p ≤ 0.05 LM-Ang II vs. LM-Saline;$ p ≤ 0.05 MyMRKO-Ang II vs. LM-Saline.

We then examined aortic endothelial stiffness by AFM in thoracic aortic explants from the different cohorts. Of note, our group has previously shown that Ang II at the dose used in this study results in aortic stiffening measured *in vivo* by pulse wave velocity (Brown et al., 2017). Ang II infusion for 4 weeks resulted in a significant increase in endothelial stiffness relative to mice infused with saline. In turn, aortic stiffness was significantly reduced relative to littermates by the abrogation of MR in myeloid cells (Figure 1B), therefore suggesting a protective effect of MyMRKO in myeloid cells on aortic endothelium.

**FIGURE 2:**
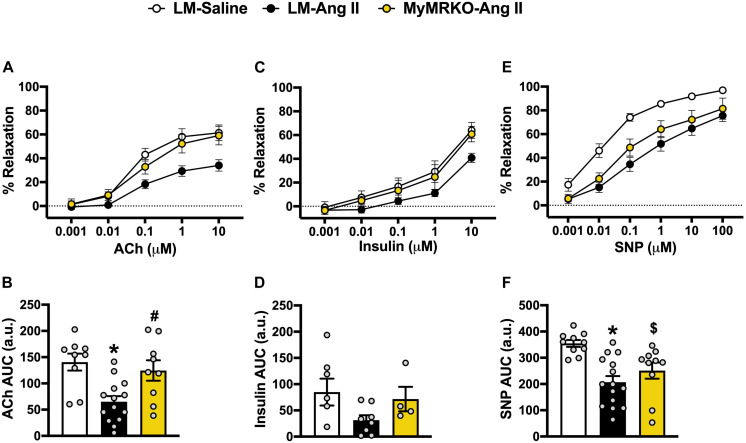
Deletion of myeloid cell MR improves endothelial function in Ang II-infused mice. Aortic vasomotor responses to acetylcholine (ACh) [**(A)** response curve, **(B)** area under the curve (AUC)], insulin **(C,D)**, and sodium nitroprusside (SNP) **(E,F)**. *p ≤ 0.05 LM-Ang II vs. LM-Saline; ^#^*p* ≤ 0.05 LM-Ang II vs. MyMRKO-Ang II; ^$^*p* ≤ 0.05 MyMRKO-Ang II vs. LM-Saline. *n* = 4–15 for all groups. LM, littermate; Ang II, angiotensin II; MyMRKO, myeloid mineralocorticoid receptor knockout.

Since vascular stiffness contributes to impaired vasodilatory function (Walker et al., 2015), we assessed aortic vasodilatory reaction in response to endothelium-dependent (ACh and insulin) and endothelium-independent (SNP) stimuli (Figure 2). Responses to preconstriction with U-46619 were not significantly different between the cohorts (prior to ACh, *p* = 0.48; prior to insulin, *p* = 0.23; prior to SNP, *p* = 0.68, data not shown). LM-Ang II mice exhibited impaired vasodilatory responses to ACh compared to the LM-saline cohort, and MR deletion in myeloid cells restored this response (Figure 2A). Similar observations were made in response to insulin; however, these effects did not reach statistical significance (Figure 2B). Ang II also resulted in impaired endothelial-independent vasorelaxation that was not significantly reverted in the MyMRKO-Ang II cohort. Collectively, these findings suggest that myeloid MR deletion improves endothelial function without affecting smooth muscle relaxation capacity in Ang II-treated mice (Figure 2C).

**FIGURE 3:**
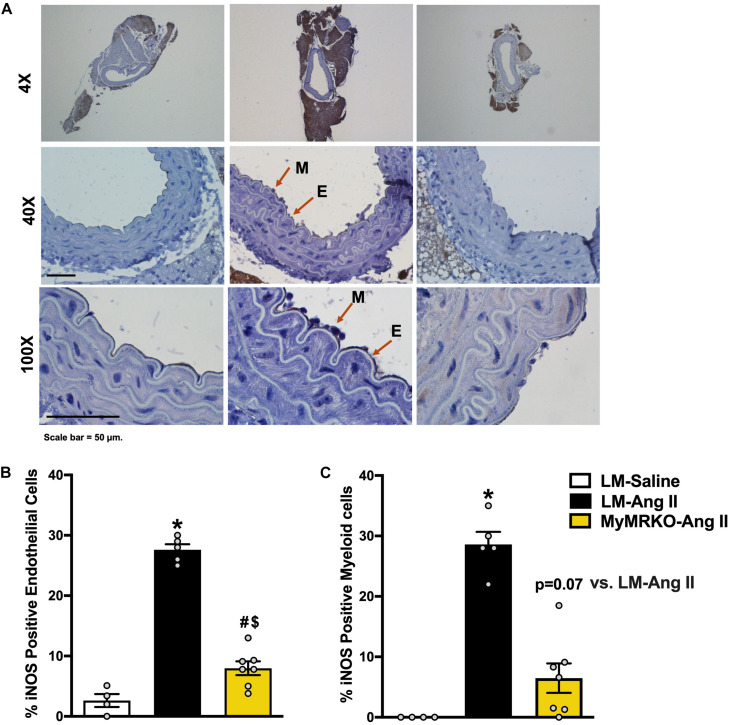
Deletion of myeloid cell MR decreases Ang II-induced iNOS expression in myeloid and endothelial cells. **(A)** Representative images of iNOS staining in myeloid and endothelial cells at 4×, 40×, and 100×of the different cohorts. **(B)** iNOS-positive endothelial cells and **(C)** iNOS-positive myeloid cells. Results are expressed as percent positive cells. *p ≤ 0.05 LM-Ang II vs. LM-Saline; ^$^*p* ≤ 0.05 MyMRKO-Ang II vs. LM-Saline; ^#^*p* ≤ 0.05 LM-Ang II vs. MyMRKO-Ang II. *n* = 4–7 for all groups. iNOS, inducible nitric oxide synthase; LM, littermate; Ang II, angiotensin II; MyMRKO, myeloid mineralocorticoid receptor knockout.

iNOS is a known marker of macrophage activation (Gunnett et al., 2005; Tejero et al., 2019), also known to be expressed in endothelial cells under inflammatory conditions (Gunnett et al., 2005; Lowry et al., 2013). Its activation can lead to endothelial dysfunction by limiting NO production by endothelial NOS (Ichihara et al., 2000; Gunnett et al., 2005; Tian et al., 2010). Herein, we examined iNOS expression in aortic tissue from the different cohorts (Figure 3A). We show that Ang II infusion results in augmented iNOS expression in endothelial and myeloid cells in the LM-Ang II cohort (Figures 3B,C). This increased expression in endothelial cells was ameliorated by myeloid MR deletion (Figures 3B,C), while there was a trend (*p* = 0.07) toward decreased expression in myeloid cells (Figure 3C).

The presence of local RAAS in VAT and in PVAT has been shown to promote oxidative stress and vascular dysfunction (Agabiti-Rosei et al., 2018). Interestingly, the phenotype of PVAT varies according to the different sections of the aorta, with the thoracic aorta region mimicking the morphology of brown adipose tissue, whereas white adipose tissue predominates in the abdominal aorta (Lian and Gollasch, 2016). Therefore, we examined the impact of MR activation in myeloid cells in response to Ang II in tPVAT, aPVAT, and VAT *via* immunohistochemistry (IHC), RT-PCR, and/or FACS.

**FIGURE 4:**
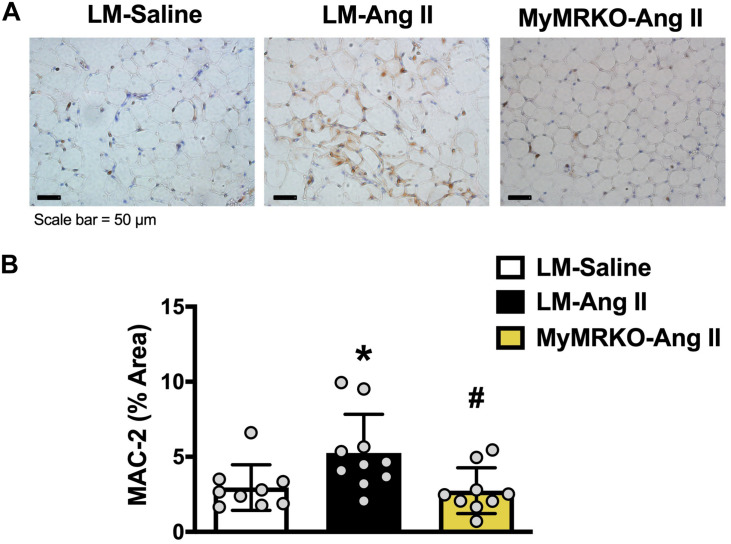
Macrophage infiltration in visceral adipose tissue is decreased in MyMRKO mice. Representative image of Mac-2 staining in visceral adipose tissue (VAT) of the different cohorts **(A)**; Mac-2 staining quantification **(B)**. *p ≤ 0.05 LM-Ang II vs. LM-Saline; ^#^*p* ≤ 0.05 LM-Ang II vs. MyMRKO-Ang II. *n* = 9–10 for all groups. LM, littermate; Ang II, angiotensin II; MyMRKO, myeloid mineralocorticoid receptor knockout.

**FIGURE 5:**
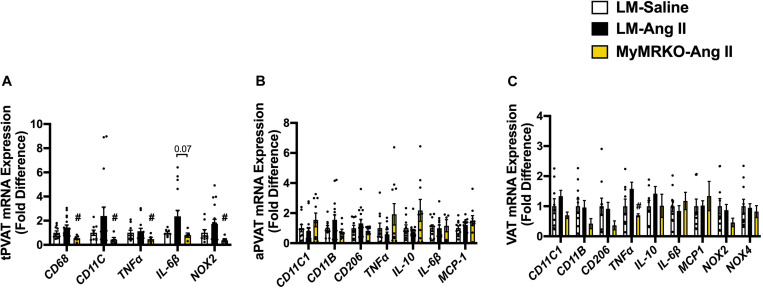
Markers of macrophage infiltration and polarization in different fat depots. **(A)** Thoracic perivascular adipose tissue (tPVAT), **(B)** abdominal perivascular adipose tissue (aPVAT), and **(C)** visceral adipose tissue (VAT). Total macrophage expression (CD11b), M1 macrophage expression (MCP-1), and M2 macrophage polarization (IL-10). ^#^*p* ≤ 0.05, LM-Ang II vs. MyMRKO-Ang II. *n* = 9–10 for all groups. LM, littermate; Ang II, angiotensin II; MyMRKO, myeloid mineralocorticoid receptor knockout.

As shown in Figure 4, Ang II resulted in increased macrophage infiltration (assessed by Mac-2 staining) compared to control in VAT, and deletion of MR in myeloid cells reversed it. Further, in tPVAT, our PCR studies showed that knockout of MR in myeloid cells caused significant reductions in markers for total macrophages as well as M1 polarization, including CD68, CD11c, NOX2, and tumor necrosis factor (TNF)-α relative to LM-Ang II, along with a trend for a reduction of interleukin (IL)-6β (Figure 5A). Conversely, no significant differences between groups or interventions were found in aPVAT (Figure 5B). In VAT, TNF-α was significantly lower in KO animals relative to littermates infused with Ang II (Figure 5C).

**FIGURE 6:**
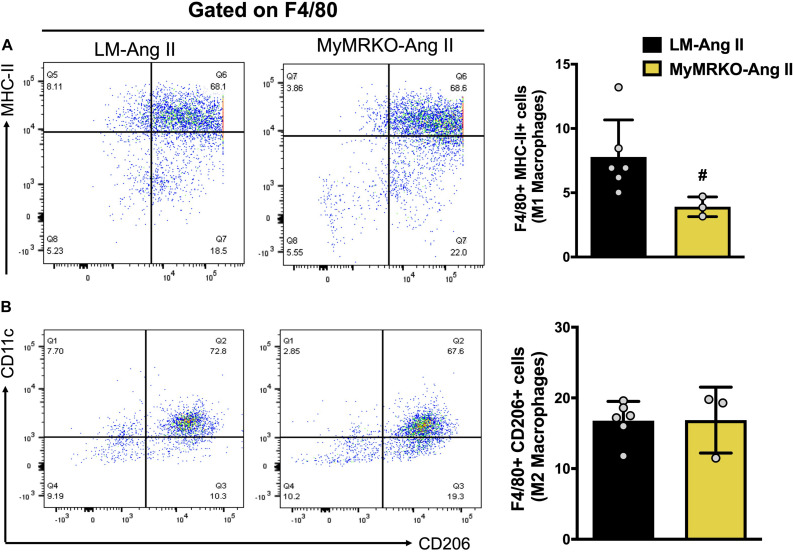
Myeloid MR deletion reduces M1 macrophage polarization in mice infused with Ang II. Flow cytometer analysis of macrophages isolated from visceral adipose tissue. **(A)** M1-macrophage polarization and **(B)** M2-macrophage polarization; ^#^*p* ≤ 0.05 LM-Ang II vs. MyMRKO-Ang II. *n* = 3–6. LM, littermate; Ang II, angiotensin II; MyMRKO, myeloid mineralocorticoid receptor knockout.

To precisely assess the impact of myeloid MR knockout in fat inflammation in a separate cohort of mice, we utilized flow cytometry to evaluate macrophage infiltration and polarization phenotype in VAT. Previous investigations have shown that Ang II infusion results in inflammation of both adipose and cardiovascular tissues (Police et al., 2009; Usher et al., 2010; Sakaue et al., 2017). Similarly, in the present investigation, we showed that Ang II infusion resulted in increased macrophage infiltration in adipose tissue when compared to the saline-treated cohort (Figure 4). To further assess the impact of myeloid MR deletion on Ang-II-induced adipose inflammation, we focused our FACS studies on characterizing the specific macrophage polarization status of VAT in mice (LM and MyMRKO) infused with Ang II. As shown in Figure 6, MyMRKO was associated with reduced infiltration/M1 macrophage polarization in response to Ang II infusion.

Collectively, our data *in vivo* and *ex vivo* show that MR abrogation in myeloid cells is protective against Ang II-induced aortic endothelial stiffening and dysfunction. These changes occurred in concert with improvements in macrophage infiltration and pro-inflammatory M1 polarization in VAT and tPVAT.

## Discussion

In the present study, we demonstrate that abrogation of MR signaling in myeloid cells in female mice protects against the development of aortic stiffening and improves endothelial function, independently of blood pressure changes and vascular smooth muscle relaxation capacity. These effects occurred in the setting of enhanced MR activation induced by Ang II infusion over 4 weeks. Furthermore, we show that vascular protection with MR deletion in myeloid cells is associated with a reduction in markers of macrophage infiltration and pro-inflammatory M1 macrophage polarization in tPVAT and in VAT. Our work expands upon previous knowledge regarding the role of immune cells as critical mediators of vascular stiffening in conditions of enhanced MR activation specifically affecting females.

Previous work has shown that MR abrogation in myeloid cells protects against cardiac and vascular inflammation, hypertrophy, and fibrosis induced by RAAS activation in male mice (Rocha et al., 2000; Rickard et al., 2009; Usher et al., 2010). Herein, we demonstrate that MyMRKO in female mice protects against aortic stiffening and improves endothelial function and in Ang II-infused mice. These changes were associated with decreased iNOS expression in the vascular wall, possibly reflecting lessened inflammation and increased NO bioavailability (Gunnett et al., 2005). However, future additional studies aimed at isolating specific cell types (e.g., myeloid cells and endothelial cells) are required to dissect the effects of MyMRKO on the recruitment of inflammatory cells and subsequent iNOS activation.

Since it has been previously demonstrated that ACh-induced aortic vasodilator responses are highly NO dependent (Chataigneau et al., 1999; Balarini et al., 2013; Crissey et al., 2015), it is reasonable to suggest that the impaired ACh and SNP vasorelaxation responses observed in our Ang II infusion model are secondary to decreased NO bioavailability and smooth muscle stiffening, as reported by others (Imanishi et al., 2006; Hong et al., 2015). Indeed, lessened NO bioavailability and Ang II exposure are known to result in aortic and vascular smooth muscle stiffening, respectively (Fitch et al., 2001). Given the extensive evidence linking adipose tissue inflammation and oxidative stress with decreased vascular NO bioavailability (Akoumianakis and Antoniades, 2017; Oikonomou and Antoniades, 2019), it is conceivable that in this model of myeloid MR deletion, decreased adipose tissue inflammation is linked to improved vascular function. It should be noted that myeloid MR deletion did not restore endothelium-independent vasodilatory responses to SNP. This suggests that the improved relaxation responses to ACh in the MyMRKO-Ang II cohort were the result of increased NO bioavailability rather than the improved capacity of smooth muscle to respond to a given concentration of NO.

In our investigation, myeloid MR deletion did not impact blood pressure elevation induced by Ang II infusion. This is similar to previous experiments in MyMRKO male mice treated with L-NAME/Ang II in which blood pressure responses were independent of other cardiovascular outcomes (Usher et al., 2010; Frieler et al., 2011; Li et al., 2014). Nevertheless, we demonstrate that myeloid MR deletion abrogates arterial stiffening induced by Ang II. This is clinically relevant given that aortic stiffening has been extensively associated with cardiovascular mortality and morbidity independently of changes in blood pressure in large cohorts (Mitchell et al., 2010; Aroor et al., 2013). Our study further extends our knowledge on the role of myeloid MR activation in mediating vascular stiffening in female mice, which have been shown to be more vulnerable to vascular dysfunction in the setting of elevated aldosterone levels when compared with males (Manrique et al., 2016; Davel et al., 2018). In this study, we did not compare females to males; however, our data support the notion that MR activation contributes to profound vascular dysfunction in females in conditions of enhanced RAAS activity such as obesity and insulin resistance (Mehta et al., 2016; Davel et al., 2018; Nayyar et al., 2018; Shukri et al., 2018).

Current literature supports a critical role for adipose tissue in the pathogenesis of CVD (Oikonomou and Antoniades, 2019) by acting as a source of inflammation that leads to dysfunction of cardiac and vascular tissues. In the present investigation, we observed a reduction in markers of macrophage infiltration and M1 polarization induced by Ang-II in female mice lacking MR in myeloid cells in VAT and tPVAT, thus expanding knowledge about the scope of MR activation on the development of vascular dysfunction in females.

Importantly, although we found improvements of Ang II-induced endothelial stiffness and endothelial function in females lacking myeloid MR along with decreased macrophage infiltration and M1 polarization in tPVAT, our data do not demonstrate a causative link. However, available literature supports a critical role for PVAT in modulating the structure and function of the underlying vasculature (Cheng et al., 2018; Qiu et al., 2018), and our findings in PVAT are in line with this possibility. Similarly, our data in VAT also suggest an impact on vascular function, since MR activation in VAT is known to play a critical role in the development of vascular stiffening and endothelial dysfunction (Nguyen Dinh Cat et al., 2016; Jia et al., 2017; Lefranc et al., 2019). Certainly, additional mechanistic studies are warranted to explore the potential mechanisms that link MR activation in specific adipose tissue compartments such as PVAT and dysfunction of the underlying vasculature.

In our adipose tissue analysis, we included VAT, tPVAT, and aPVAT. tPVAT is morphologically and functionally different from aPVAT and VAT, as it expresses genes specific to brown adipose tissue that promote thermogenesis, lipid oxidation, and insulin sensitivity (Fitzgibbons et al., 2011). Furthermore, the presence of immune cells, in particular macrophages, is notoriously lower in tPVAT than VAT and aPVAT (Fitzgibbons et al., 2011; Padilla et al., 2013). These features highlight a protective role of tPVAT under physiologic conditions (Nosalski and Guzik, 2017; Horimatsu et al., 2018). Although our study does not focus on detecting “browning” or “whitening” changes on adipose tissue, we speculate that the Ang II impact can be at least partially related to a myeloid MR-driven shift toward a white adipose tissue phenotype. Indeed, “whitening” of adipose tissue has been described in response to different stimuli capable of inducing macrophage infiltration (Kotzbeck et al., 2018), such as Ang II (Kawabe et al., 2019). In line with our findings, other investigators have reported that Ang II infusion in mice also results in stiffening of the abdominal aorta (Tham et al., 2002), which is surrounded by aPVAT. Therefore, it is likely that our findings in the thoracic aorta also occurred in the abdominal aorta. Although our findings using PCR were not statistically significant, we demonstrate a protective effect of MR KO in myeloid cells against Ang II-induced macrophage infiltration/M1 polarization in VAT. This apparent disparity can be explained by the use of IHC and flow cytometry, which more specifically targets macrophage markers in VAT, whereas PCR was utilized to analyze whole aPVAT tissue.

A limitation of the MyMRKO model is that the KO affects the whole myeloid lineage and not only macrophages. Available data in this regard are still sparse; however, the degree of MR abrogation specifically in macrophages appears to be predominant over other types of myeloid cells (Usher et al., 2010) and has been reported to be as high as 80% in macrophages, whereas it is significantly lower (approximately 50%) in circulating neutrophils (Huang et al., 2014). Another confounding factor inherent to the MyMRKO model is that MR expression is abrogated in myeloid cells independently of their origin and not exclusively in adipose tissue. Therefore, we cannot exclude an impact of MR abrogation in other tissues on our findings in the vasculature. Also, the impact of MRKO is variable in different cell types. While there is incomplete abrogation in resident peritoneal macrophages (30–40%) as well in the brain (Cho et al., 2008), there is near-absolute abolition of MR expression in heart tissue (Usher et al., 2010). In addition, it is also important to highlight that macrophages do not express 11β-hydroxysteroid dehydrogenase type 2, the enzyme that inactivates glucocorticoids and provides specificity to mineralocorticoid actions. Therefore, MR activation in macrophages is likely to be mediated instead by glucocorticoids (Rickard et al., 2009; Usher et al., 2010), although direct MR activation by Ang II has been described (Lu et al., 2019). Despite these limitations of the MyMRKO model, our findings support a critical role for myeloid cells, in particular macrophages, as potential mediators of vascular injury resulting from inappropriate MR activation.

Collectively, our study adds to mounting evidence in both preclinical models and clinical studies demonstrating a critical role of inappropriate MR activation and enhanced mineralocorticoid activity that profoundly affects vascular function in females and potentially contributes to more severe CVD relative to males (Manrique et al., 2016; Mehta et al., 2016; Lastra et al., 2017). In the present study, we demonstrate that MR activation in myeloid cells is critical to the development of vascular stiffness and abnormal vasomotor responses in a female mouse model of MR activation. Further, our data suggest that enhanced MR signaling predominantly in macrophages promotes vascular stiffness and endothelial dysfunction in concert with enhanced M1 macrophage polarization and inflammation in VAT as well as PVAT. These novel data add to current knowledge about the role played by MR activation in myeloid cells in the pathogenesis of vascular dysfunction and contribute to a better understanding of the pathophysiology of CVD in females, who are disproportionately more affected in conditions of RAAS activation relative to men.

## Data Availability Statement

The raw data supporting the conclusions of this article will be made available by the authors, without undue reservation.

## Ethics Statement

The animal study was reviewed and approved by Institutional Animal Use and Care Committee (IACUC) at the University of Missouri-Columbia.

## Author Contributions

All authors listed have made a substantial, direct and intellectual contribution to the work, and approved it for publication.

## Conflict of Interest

The authors declare that the research was conducted in the absence of any commercial or financial relationships that could be construed as a potential conflict of interest.
